# Predictors of Reamputation in Patients With Advanced‐Stage Thromboangiitis Obliterans Ulcers: A Retrospective Cohort Study

**DOI:** 10.1111/iwj.70853

**Published:** 2026-02-18

**Authors:** Yiğit Önaloğlu, Ömer Faruk Gümüş, Bekir Karakılıç, Ali Volkan Özlük, Emin Can Balcı, Mehmet Ali Talmaç, Melih Civan

**Affiliations:** ^1^ Department of Orthopedics and Traumatology Başakşehir Çam and Sakura City Hospital Istanbul Türkiye; ^2^ Department of Orthopedics and Traumatology Bagcilar Training and Research Hospital Istanbul Türkiye

**Keywords:** amputation, foot ulcer, thromboangiitis obliterans, tobacco use, treatment outcome

## Abstract

Thromboangiitis obliterans (TAO) is a rare, tobacco‐associated vasculitis that primarily affects the distal extremities of young males. In advanced stages, it often leads to chronic limb ischemia with ischemic ulceration, culminating in amputation. Data on risk factors for reamputation in this population remain limited. This study aimed to identify clinical, radiological and microbiological predictors of reamputation in patients with TAO‐related foot ulcers undergoing amputation. A retrospective cohort study was conducted on 25 patients (31 limbs) with Fontaine stage IV TAO ulcers who underwent lower extremity amputation between January 2021 and December 2024. Patients were stratified into two groups based on whether they underwent repeat amputation (Group 1) or a single procedure (Group 2). Preoperative magnetic resonance imaging, intraoperative tissue cultures and laboratory data were evaluated. Smoking status, hospitalisation metrics and adjunctive therapies were recorded. Statistical analysis included Mann–Whitney U, Fisher's exact test, ROC curve analysis and multivariate logistic regression. Seventeen limbs required reamputation. Persistent smoking was observed in 100% of Group 1 compared with 58.4% of Group 2 (*p* = 0.015). Positive intraoperative cultures were significantly more frequent in Group 1 (64.7% vs. 21.4%; *p* = 0.029), with all multidrug‐resistant organisms confined to this group. Length of hospital stay was significantly longer in Group 1 (25.2 ± 6.4 vs. 15.8 ± 5.3 days; *p* = 0.001). ROC analysis identified > 19 days of hospitalisation as a threshold for reamputation risk (AUC = 0.781; *p* = 0.018). Multivariate analysis identified three independent predictors of reamputation: persistent smoking (OR: 5.2, 95% CI: 1.2–22.8; *p* = 0.015), positive intraoperative culture (OR: 4.7, 95% CI: 1.1–20.1; *p* = 0.041), and hospitalisation longer than 19 days (OR: 6.5, 95% CI: 1.4–29.4; *p* = 0.018). Reamputation in advanced‐stage TAO is strongly associated with modifiable factors, particularly ongoing tobacco use, Gram‐negative infection and prolonged hospital stay. Early identification and targeted intervention addressing these variables may improve limb preservation outcomes in this high‐risk population.

## Key Points

- Persistent smoking was an independent predictor of reamputation in advanced‐stage TAO (OR 5.2).
- Positive intraoperative cultures—particularly multidrug‐resistant Gram‐negative organisms—were strongly associated with repeat amputation.
- Hospitalisation longer than 19 days demonstrated good discriminatory ability for reamputation risk (AUC 0.781).
- Reamputation in TAO is driven by modifiable behavioural and infectious factors rather than radiological findings alone.
- Early smoking cessation and culture‐directed therapy may improve limb preservation outcomes in this high‐risk population.

## 1 Introduction

Thromboangiitis obliterans (TAO), or Buerger's disease, is an uncommon inflammatory vasculopathy that affects small‐ and medium‐sized arteries and veins in the extremities. It primarily occurs in younger male individuals with a history of tobacco use. The disease is marked by segmental inflammation, thrombosis and progressive occlusion of peripheral vessels.

Unlike ulcers associated with diabetes or atherosclerotic peripheral arterial disease (PAD), those caused by TAO often follow an unpredictable course and are typically resistant to standard revascularisation procedures and limb preservation strategies [1]. In its advanced stages, TAO can result in chronic limb ischemia characterised by ischemic ulceration, tissue necrosis and eventual limb loss. Longitudinal studies have shown that up to one‐third of patients with TAO may require an amputation within 15 years of diagnosis [2].

Tobacco exposure remains the most important modifiable factor in the pathogenesis of TAO. Complete smoking cessation is currently the only intervention proven to halt disease progression [3]. Despite this, long‐term abstinence remains difficult to achieve, and the lack of effective alternative therapies often leads to progressive ischemia and poor surgical outcomes. Beyond tobacco exposure, emerging evidence suggests that genetic susceptibility and immune‐mediated mechanisms may contribute to TAO pathogenesis. Associations with specific HLA haplotypes and dysregulated inflammatory responses have been reported [4]. In addition, periodontal bacterial infection—particularly involving *Porphyromonas gingivalis* —has been proposed as a potential trigger through molecular mimicry and endothelial injury, suggesting that TAO may represent a systemic inflammatory disorder rather than a purely localised vascular disease [5].

Although risk factors for reamputation have been well studied in populations with diabetic foot ulcers (DFUs) and atherosclerotic PAD [6], data specific to TAO remain sparse. Given that TAO often affects younger individuals, repeated surgical interventions can result in substantial physical, emotional and socioeconomic burdens.

Given that thromboangiitis obliterans predominantly affects individuals in the economically active age group, repeat amputations impose a substantial socioeconomic burden. These include prolonged work disability, loss of employment, long‐term prosthetic dependence, psychological distress and increased healthcare utilisation. Unlike diabetic or elderly peripheral arterial disease populations, limb loss in TAO frequently results in decades of reduced productivity and social participation, underscoring the importance of identifying preventable risk factors for reamputation.

This study aimed to identify clinical, microbiological, and radiological predictors of reamputation in patients presenting with advanced‐stage TAO‐related foot ulcers. By clarifying these factors, clinicians may be better equipped to individualise treatment strategies and improve limb salvage in this challenging patient population.

## 2 Materials and Methods

This retrospective cohort study was approved by the Institutional Ethics Committee (protocol number 2025‐137, approval date June 3, 2025) and was conducted in accordance with the 1975 Declaration of Helsinki. Written and verbal informed consent was obtained from all patients prior to their inclusion in the study.

**FIGURE 1:**
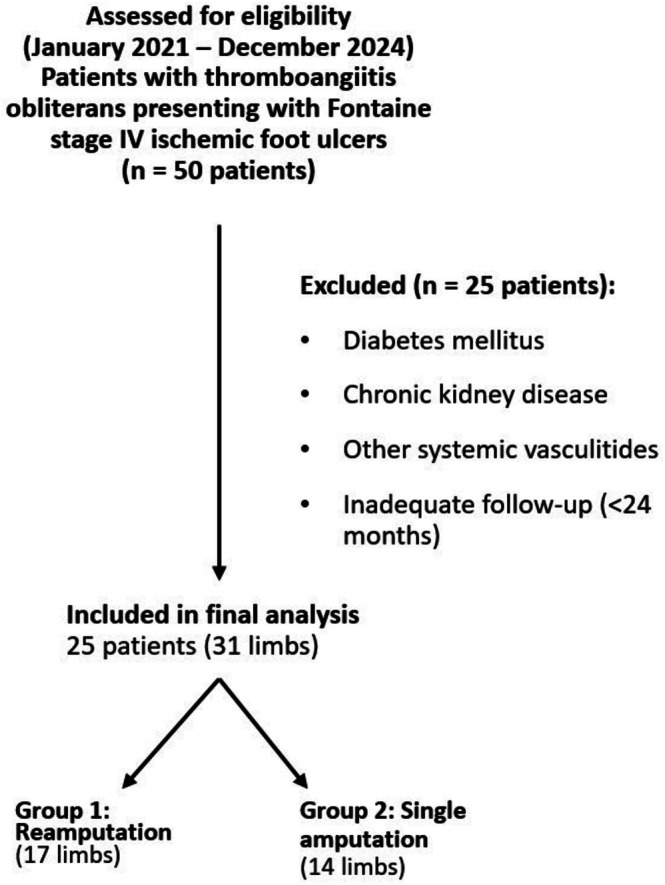
Flow diagram of patient selection and cohort formation. Between January 2021 and December 2024, patients with thromboangiitis obliterans presenting with Fontaine stage IV ischemic foot ulcers were assessed for eligibility. After application of predefined exclusion criteria, 25 patients (31 limbs) were included in the final analysis and stratified into the reamputation group (Group 1, 17 limbs) and the single amputation group (Group 2, 14 limbs).

Between January 2021 and December 2024, a total of 25 patients (31 limbs) diagnosed with TAO and presenting with Fontaine stage IV ischemic foot ulcers underwent lower extremity amputation at a single tertiary referral centre. The diagnosis of TAO was established based on widely accepted clinical criteria: a history of tobacco use, distal arterial occlusions in the extremities, absence of major atherosclerotic risk factors, and segmental occlusions on angiographic imaging. Patients with diabetes mellitus, chronic kidney disease, or other systemic vasculitides were excluded. Only those with a minimum of 24 months of postoperative follow‐up were included in the final analysis. A flow diagram illustrating patient selection, exclusion criteria and final cohort allocation is presented in Figure 1.

Magnetic resonance imaging (MRI) was performed using a 1.5‐Tesla system, incorporating STIR, T1‐weighted, and T2‐weighted sequences to evaluate for osteomyelitis. All patients received medical management tailored to TAO, including vasodilators, calcium channel blockers, and antiplatelet agents supervised by a cardiovascular surgeon.

Smoking status was recorded during each outpatient visit and based on patient self‐report. Vascular assessments and endovascular treatment planning were conducted jointly by interventional radiology and cardiovascular surgery departments. All patients received empiric intravenous antibiotics prior to surgery, which were later modified according to intraoperative deep tissue culture results and antimicrobial susceptibility profiles.

Advanced wound care modalities such as negative pressure wound therapy, skin grafting or reconstructive flap procedures were not employed due to extensive ischemia, poor distal perfusion, active infection and limited tissue viability, rendering limb salvage strategies unfeasible in most cases.

Amputation levels were determined through multidisciplinary consensus involving orthopaedic, cardiovascular, plastic and reconstructive surgery and infectious disease teams. Surgical planning was informed by clinical findings, imaging results, and an intraoperative assessment of tissue viability. Orthopaedic surgeons experienced in lower limb amputations performed the procedures, ensuring consistency in surgical approach.

Amputations were classified as:

- Minor amputations: procedures distal to the ankle, including toe, ray and transmetatarsal resections.
- Major amputations: those at or above the Syme level, including below‐knee or above‐knee amputations.

Patients were stratified into two groups:

- Group 1: those who required one or more reamputations following the index surgery.
- Group 2: those who underwent a single amputation without further bony intervention.

Reamputation was defined as any secondary surgical procedure involving additional bone resection following the initial amputation. Soft tissue revisions that did not require osseous resection were not classified as reamputations.

The following clinical parameters were examined:

- Age, sex and comorbidity status
- Smoking status and cessation
- Preoperative serum C‐reactive protein (CRP) levels (measured within 48 h before surgery)
- MRI‐confirmed osteomyelitis
- Intraoperative tissue culture results
- Use of hyperbaric oxygen therapy (HBOT)
- Length of initial hospital stay
- Number of hospital readmissions during follow‐up

Data were collected from institutional electronic medical records. Smoking status was assessed based on patient self‐report during outpatient follow‐up, without biochemical validation. Statistical analysis was performed using IBM SPSS Statistics for Windows, version 28.0 (IBM Corp., Armonk, NY, USA). Normality was assessed using the Shapiro–Wilk test. Continuous variables were analysed using the Mann–Whitney U test. Categorical variables were assessed using Chi‐square or Fisher's exact test, depending on cell size.

Receiver operating characteristic (ROC) curve analysis was used to assess the predictive performance of hospitalisation duration for reamputation. Area under the curve (AUC), sensitivity, and specificity were reported. Multivariate logistic regression was performed to identify independent predictors of reamputation. Variables with *p* < 0.1 in univariate analysis or deemed clinically significant were included in the model. Odds ratios (ORs) and 95% confidence intervals (CIs) were calculated. Model calibration was evaluated using the Hosmer–Lemeshow goodness‐of‐fit test.

## 3 Results

A total of 31 limbs from 25 patients were evaluated. Bilateral lower limb amputations were observed in six patients, corresponding to a rate of 30.8% in Group 1 (4 of 13 patients) and 16.7% in Group 2 (2 of 12 patients). The remaining patients underwent unilateral amputation. When bilateral cases were analysed per limb, 17 limbs were assigned to Group 1 (reamputation group) and 14 to Group 2 (single amputation group). Group‐wise demographic, clinical and microbiological characteristics are summarised in Table 1.

**TABLE 1:** Comparison of demographic, clinical, microbiological and perioperative variables between reamputation (Group 1) and single amputation (Group 2) groups.

Variable	Group 1 (Reamputation)	Group 2 (Single amputation)	*p*
Number of patients	13	12	—
Number of limbs	17	14	—
Mean age (years)	59.6 ± 17.2	62.8 ± 18.1	0.810
Male sex (%)	92.3	58.3	0.073
Bilateral lower limb involvement (%)	30.8	16.7	0.642
Preop endovascular intervention (%)	29.4	35.7	1.000
Smoking cessation (%)	0	41.6	0.015*
Minor amputations (initial)	10	7	0.725
Major amputations (initial)	7	7	0.725
Additional amputations (major/minor)	15/2	0/0	—
Time to reamputation (days, mean ± SD)	175.4 ± 129.3	—	—
MRI‐confirmed foot osteomyelitis (%)	58.8	57.1	1.000
Preoperative CRP (mg/L, median [IQR])	47.2 (28.6–71.4)	23.0 (12.4–39.8)	0.807
Positive intraoperative cultures (%)	64.7	21.4	0.029*
HBOT received (%)	23.1	0	0.220
Hospitalisation (days, mean ± SD)	25.2 ± 6.4	15.8 ± 5.3	0.001*
Hospital readmissions (mean ± SD)	1.71 ± 0.6	1.06 ± 0.25	0.001*

*Note:* Categorical variables were compared using Fisher's exact test. Due to non‐parametric distribution, continuous variables were assessed using the Mann–Whitney U test. A *p‐value of less than 0.05 was considered statistically significant. The Bold values presented in Table 1 indicate statistically significant differences between the groups (*p* < 0.05).

No statistically significant differences were identified between the two groups with respect to mean age (59.6 ± 17.2 vs. 62.8 ± 18.1 years; *p* = 0.810), male sex distribution (92.3% vs. 58.3%; *p* = 0.073), bilateral limb involvement (30.8% vs. 16.7%; *p* = 0.642), or preoperative endovascular intervention rates (29.4% vs. 35.7%; *p* = 1.000).

Smoking cessation was achieved in none of the patients in Group 1, whereas 41.6% of Group 2 patients had discontinued tobacco use (*p* = 0.015). The index amputations in Group 1 included 10 minor and 7 major procedures, whereas Group 2 comprised 7 minor and 7 major amputations (minor: *p* = 0.725; major: *p* = 0.725). All Group 1 patients subsequently underwent additional amputations, totalling 17 reinterventions (2 minor, 15 major). The mean duration from the initial surgery to the second amputation was 175.4 ± 129.3 days.

MRI‐confirmed foot osteomyelitis was detected in 58.8% of limbs in Group 1 and 57.1% in Group 2 (*p* = 1.000). Preoperative serum CRP levels were higher in Group 1 (median [IQR]: 47.2 [28.6–71.4] mg/L) compared to Group 2 (23.0 [12.4–39.8] mg/L), although this difference did not reach statistical significance (*p* = 0.807).

Positive intraoperative deep tissue cultures were significantly more common in Group 1 (64.7%) than in Group 2 (21.4%) (*p* = 0.029). All observed polymicrobial infections occurred in Group 1 (3 limbs, 17.6%), with cultured organisms including *Escherichia coli* , *Pseudomonas aeruginosa* , *Enterobacter cloacae* , *Acinetobacter baumannii* and *Corynebacterium striatum* . Gram‐negative pathogens predominated in Group 1 (52.9%), whereas Group 2 primarily exhibited Gram‐positive species (21.4%), such as *Staphylococcus aureus* and *Enterococcus faecalis* . No bacterial growth was observed in 35.3% of Group 1 limbs and 78.6% of Group 2 limbs.

Multidrug‐resistant organisms (MDROs) were identified exclusively in Group 1. These included extended‐spectrum beta‐lactamase (ESBL)‐producing strains of *Escherichia coli* , *Pseudomonas aeruginosa* , *Enterobacter cloacae* and *Acinetobacter baumannii* . Of the positive cultures in Group 1, 45.5% (5 of 11) were multidrug‐resistant Gram‐negative isolates. In contrast, all bacterial isolates from Group 2 were susceptible Gram‐positive organisms. Polymicrobial infections involving MDROs were observed in 17.6% of limbs in Group 1, with no such cases in Group 2.

HBOT was administered to 23.1% of patients in Group 1, whereas none of the patients in Group 2 received this therapy (*p* = 0.220).

Group 1 exhibited significantly longer hospital stays (25.2 ± 6.4 days vs. 15.8 ± 5.3 days; *p* = 0.001) and a higher mean readmission frequency (1.71 ± 0.6 vs. 1.06 ± 0.25; *p* = 0.001).

**FIGURE 2:**
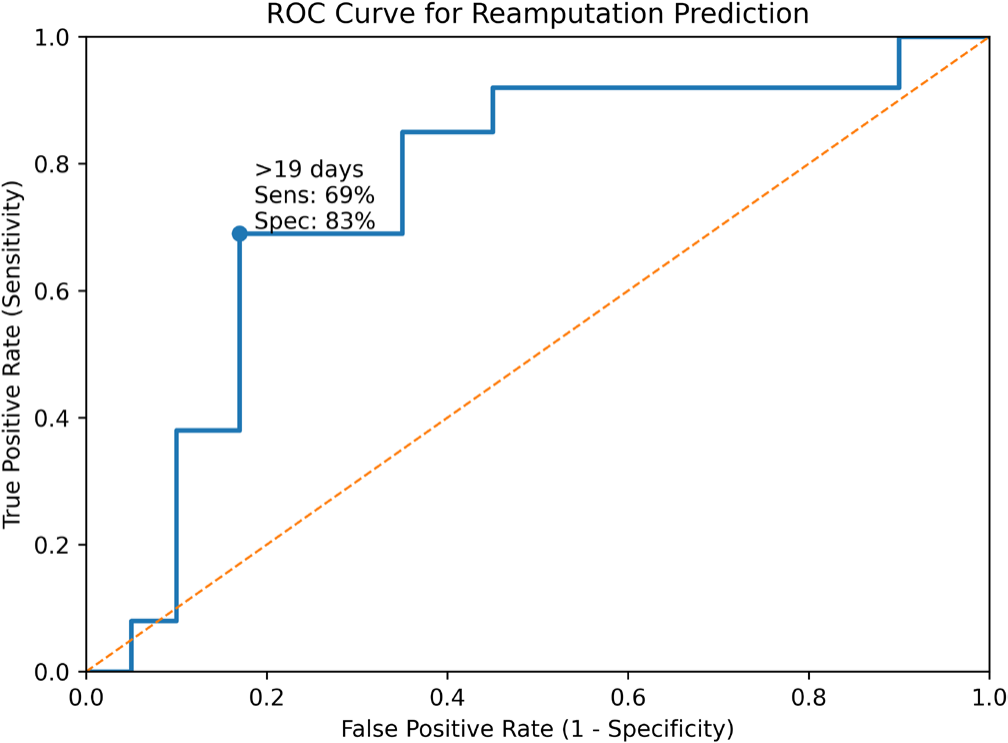
Receiver operating characteristic (ROC) curve analysis for reamputation prediction based on hospitalisation duration. A threshold of > 19 days yielded an area under the curve (AUC) of 0.781 (95% CI: 0.612–0.949; *p* = 0.018), with 69% sensitivity and 83% specificity.

Receiver operating characteristic (ROC) curve analysis identified a hospitalisation duration of > 19 days as a significant predictor of reamputation, yielding an area under the curve (AUC) of 0.781 (95% CI: 0.612–0.949; *p* = 0.018). This threshold demonstrated 69% sensitivity and 83% specificity for predicting repeat amputation (Figure 2).

The logistic regression model demonstrated acceptable calibration (Hosmer–Lemeshow *p* > 0.05), supporting the robustness of the multivariate findings. The results of the multivariate logistic regression analysis identifying independent predictors of reamputation are presented in Table 2.

**TABLE 2:** Multivariate logistic regression analysis identifying independent predictors of reamputation.

Variable	OR	95% CI	*p*
Persistent smoking	5.2	1.2–22.8	0.015
Positive intraoperative culture	4.7	1.1–20.1	0.041
Hospital stay > 19 days	6.5	1.4–29.4	0.018

*Note:* Multivariate logistic regression analysis was performed to identify independent predictors of reamputation. Variables with *p* < 0.10 in univariate analysis and those considered clinically relevant were included in the model. Odds ratios (ORs) are presented with 95% confidence intervals (CIs). A *p*‐value < 0.05 was considered statistically significant.

## 4 Discussion

This study underscores the multifactorial nature of reamputation in patients with advanced‐stage TAO‐related ulcers. Despite standardised surgical protocols, a significant number of individuals underwent subsequent amputations. The analysis identified three independent predictors of reamputation: persistent smoking, positive intraoperative cultures and prolonged hospitalisation. These findings highlight the complex interaction between behavioural, infectious, and perioperative variables in determining surgical outcomes in TAO, a disease often resistant to conventional revascularisation and wound healing interventions.

### 4.1 Tobacco Use as a Central Determinant of Outcome

Tobacco exposure remains a pivotal element in TAO pathogenesis. In our cohort, no patient in the reamputation group had ceased smoking, whereas 41.6% of those in the single amputation group reported successful cessation (*p* = 0.015). Persistent tobacco use was independently predictive of reamputation (OR: 5.2; 95% CI: 1.2–22.8), reinforcing previous evidence that smoking cessation is the only intervention consistently associated with disease stabilisation [7, 8]. Nicotine induces vasoconstriction, disrupts endothelial integrity and impairs neovascularisation, all of which contribute to tissue hypoxia and poor wound healing [9]. These results support embedding structured cessation support within comprehensive TAO care pathways.

### 4.2 Infectious Burden and Its Role in Surgical Failure

Positive intraoperative cultures were significantly more common in the reamputation group than in the single‐amputation group (64.7% vs. 21.4%; *p* = 0.029). Notably, all cases of polymicrobial infection and multidrug‐resistant organism (MDRO) isolation occurred exclusively in the reamputation group. The predominant MDROs included extended‐spectrum beta‐lactamase (ESBL)‐producing *Escherichia coli* , *Pseudomonas aeruginosa* , *Enterobacter cloacae* and *Acinetobacter baumannii* —organisms characterised by enhanced virulence, biofilm production and high antimicrobial resistance. These traits are known to delay wound resolution and increase the likelihood of surgical failure [10, 11]. In contrast, patients in the single‐amputation group yielded more treatment‐responsive organisms such as *Staphylococcus aureus* and *Enterococcus faecalis* [12, 13]. Similar to patterns observed in DFU and PAD literature, the presence of Gram‐negative pathogens has been associated with worse clinical outcomes and higher amputation rates [14, 15, 16, 17]. Our findings align with these trends, highlighting the necessity for early debridement, infectious disease collaboration and culture‐directed antimicrobial therapy in managing TAO‐related infections.

### 4.3 Amputation Level and Surgical Planning

Most patients in Group 1 who initially underwent minor amputations eventually required progression to major procedures, indicating that limited surgical resection may be inadequate in the setting of advanced ischemia. This trend is echoed in both DFU and PAD cohorts, where insufficient debridement has been associated with poorer limb salvage [18, 19]. Although MRI‐confirmed osteomyelitis did not differ significantly between groups (58.8% vs. 57.1%), its presence remains a critical factor in surgical planning. Residual bone infection is a well‐known contributor to delayed healing and surgical failure, even when prolonged antibiotic therapy is employed [20, 21]. Preoperative imaging and intraoperative assessment can help optimise resection margins and prevent further tissue loss.

### 4.4 Systemic Inflammation and CRP Utility

Although the mean CRP value was higher in Group 1 (47.2 vs. 23.0 mg/L), the difference was not statistically significant (*p* = 0.807), possibly due to inter‐patient variability or sample size limitations. In other chronic wound populations, elevated CRP has been linked to worse healing outcomes, increased risk of reamputation and mortality [22, 23, 24, 25]. However, its utility as a prognostic marker in TAO remains uncertain and likely requires disease‐specific interpretation [26]. Nevertheless, CRP could still serve as an adjunct in assessing systemic inflammatory status and infection severity.

### 4.5 Adjunctive Therapies and Perioperative Support

HBOT was employed only in the reamputation group (23.1%), suggesting its use as a salvage intervention in severe or refractory cases. Although HBOT has demonstrated efficacy in ischemic and diabetic ulcers, its role in TAO‐specific ulcers is less clearly defined [27, 28, 29, 30]. Notably, none of the patients received NPWT, which has been shown to facilitate granulation and reduce microbial burden in other chronic wound settings [31, 32]. Earlier application of these adjunctive modalities in high‐risk patients may reduce the likelihood of reintervention.

### 4.6 Length of Hospital Stay as a Prognostic Indicator

Hospitalisation more than 19 days was an independent risk factor for reamputation (OR: 6.5; 95% CI: 1.4–29.4; *p* = 0.018), supported by ROC analysis demonstrating good discriminatory power (AUC = 0.781). Prolonged hospital stay may reflect a more severe clinical course, delayed healing, or increased need for perioperative interventions. Although readmission rates were also significantly higher in Group 1 (1.71 vs. 1.06; *p* = 0.001), they did not independently predict reamputation. These results are consistent with existing DFU literature and support coordinated inpatient care to minimise extended hospitalisation and its associated risks [33, 34].

### 4.7 Innovative and Regenerative Therapies

Despite consistent use of standard surgical and medical treatments, over half of the patients required reamputation, highlighting the limitations of conventional approaches in advanced TAO. Emerging regenerative strategies, including autologous stem cell transplantation and gene therapies promoting angiogenesis (e.g., VEGF, FGF), offer promise in cases of non‐reconstructable ischemia [35, 36]. Although preliminary outcomes are encouraging, robust clinical trials focused on TAO populations are needed to confirm their efficacy and define optimal application protocols.

## 5 Study Limitations

This study has notable limitations. Its retrospective nature precludes definitive causal inference and may introduce selection bias. The small cohort size reduces the statistical power of subgroup analyses and limits generalisability. Smoking status was based on self‐reported data without biochemical confirmation, which may have resulted in misclassification. Although imaging and microbiological assessments were systematically performed, variability in intraoperative sampling and clinical decision‐making may have influenced surgical outcomes. Functional outcomes such as ambulatory status, prosthesis use and quality‐of‐life measures were not assessed and represent important endpoints for future studies.

Given the rarity of thromboangiitis obliterans, high‐quality comparative data remain scarce. Therefore, this discussion incorporates references from diabetic foot and peripheral arterial disease studies to provide context. Although such comparisons are clinically valuable, caution is warranted due to differences in disease pathophysiology and response to treatment. Larger, prospective multicenter studies are required to validate these findings and enhance their applicability in real‐world practice.

## 6 Conclusion

Reamputation in patients with advanced‐stage TAO‐related ulcers is closely linked to modifiable behavioural, infectious and perioperative factors. This study identified persistent smoking, microbiologically confirmed infection—particularly with multidrug‐resistant Gram‐negative pathogens—and hospital stays more than 19 days as independent predictors of surgical failure. These results reinforce the multifactorial nature of limb loss in TAO and highlight the need for comprehensive, individualised treatment strategies.

To improve limb salvage in this high‐risk population, clinical management should prioritise early and sustained smoking cessation, prompt surgical debridement and antibiotic regimens guided by intraoperative culture results. Minimising hospitalisation duration through efficient multidisciplinary care may further reduce reamputation risk. As conventional treatment approaches remain limited in efficacy, future research should explore the integration of regenerative modalities and refine predictive tools to enhance patient outcomes and reduce the burden of repeat amputation in TAO.

## Funding

The authors have nothing to report.

## Ethics Statement

This retrospective cohort study was approved by the Institutional Ethics Committee (protocol number 2025‐137, approval date June 3, 2025) and was conducted in accordance with the principles of the 1975 Declaration of Helsinki.

## Consent

Written and verbal informed consent was obtained from all patients prior to inclusion in the study.

## Conflicts of Interest

The authors declare no conflicts of interest.

## Data Availability

The data that support the findings of this study are available on request from the corresponding author. The data are not publicly available due to privacy or ethical restrictions.
